# Smart Composites and Processing

**DOI:** 10.3390/polym14194166

**Published:** 2022-10-04

**Authors:** Kwang-Jea Kim

**Affiliations:** DTR VMS Italy S.r.l., Via S. Antonio n. 59, Passirano, 25050 Brescia, Italy ; kwangjea.kim@gmail.com

Polymer composites have been at the forefront of research in recent decades as a result of the unique properties they provide for utilization in numerous applications.

The factors affecting the manufacturing of a smart composite includes the choice of ingredients such as polymer, filler and additives as well as their unique composition [1]. These include polymers (modification, blending, general/engineering/super engineering plastic), rubber (natural rubber (NR), EPDM, silicone, TPE, specialty rubber), fibers (size, shape, dispersion/distribution, reinforcement, electrical/thermal conduction, biodegradable, cellulose-, glass-, carbon-, graphene-, aramid-, etc.), and additives (synergistic/antagonistic, anti-degradation, interfacial-adhesion, silane hybrid composites) [2,3]. An example of interfacial adhesion is bifunctional silane [e.g., bis(triethoxysilyilpropyl)disulfide (TESPT) and bis(triethoxysilyilpropyl)tetrasulfide (TESPD)] in a silica-filled rubber system. In the silica filled system, bifunctional silane chemically bonds silica and NR, and not only assists in the dispersion of silica agglomerates in the rubber chain [4,5], but also increases the interfacial interaction between the two materials by chemical bonding [6].

Figure 1 shows the TESPT effect on chemical bonding between silica and NR. Silanes represent smart materials, which change the properties of rubber and plastic composites significantly, as shown above. It was applied for smart/green tire composites, anti-vibration rubber composites, and plastic composites [7,8].

Additionally, the smart processing of polymer composites improves the construction of polymer composites, which is influenced by the choice of mixers, processing condition, processing technique, use of a 3D printer, etc. These include the mixing mechanisms [mixer (internal/open), intermeshing/tangential type (rotor type and screw configuration), reactive mixing (temperature/speed at each stage, curing condition (pre/post), mixing sequence, mold design and molding technique (filler orientation, simulation, pre-treatment (chemical, thermal…)) etc.] [4,5,9,10,11,12,13].

These contribute to the construction of advanced polymer composites for high-performance automotive and aerospace parts, advanced electronic devices, environmentally friendly goods, sensors, and other such uses.

“Smart Composites and Processing” is a newly opened Special Issue (SI) of Polymers, which aims to publish original and review papers on the new scientific and applied research and make boundless contributions to the findings and understanding of various smart composites and smart processing.

## Figures and Tables

**Figure 1:**
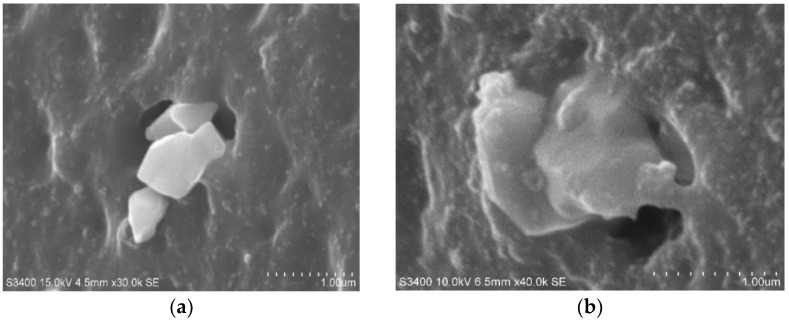
SEM photograph of silica-NR compound (**a**) without silane, (**b**) with silane (TESPT) [6].

## References

[1] White J.L., Kim K.J. (2008). Thermoplastic and Rubber Compounds Technology and Physical Chemistry.

[2] Plueddemann E.P. (1982). Silane Coupling Agents.

[3] Gupta R.K., Kennal E., Kim K.J. (2009). Polymer Nanocomposites Handbook.

[4] Kim K.J., White J.L. (2000). Silica Agglomerate Breakdown in Three-Stage Mix Including a Continuous Ultrasonic Extrude. J. Ind. Eng. Chem..

[5] Kim K.J., White J.L. (2001). TESPT and Different Aliphatic Silane Treated Silica Compounds Effects on Silica Agglomerate Dispersion and on Processability During Mixing in EPDM. J. Ind. Eng. Chem..

[6] Kim K.J. (2013). Silane effects on in-rubber silica dispersion and silica structure (α(F)): A Review. Asian J. Chem..

[7] Ryu C.S., Kim K.J. (2022). Interfacial Adhesion in Silica-Silane Filled NR Composites: A Short Review. Polymers.

[8] Lee J.Y., Kim K.J. (2019). MEG effects on hydrolysis of polyamide 66/glass fiber composites and mechanical property changes. Molecules.

[9] Wolff S. (1981). Reinforcing and Vulcanization Effects of Silane Si69 in Silica-Filled Compounds. Kautsch. Gummi Kunstst..

[10] Wolff S. (1982). Optimization of Silane-Silica OTR Compounds. Part 1: Variations of Mixing Temperature and Time during the Modification of Silica with Bis-(3-Triethoxisilylpropyl)-Tetrasulfide. Rubber Chem. Technol..

[11] Isayev A.I., Hong C.K., Kim K.J. (2003). Continuous Mixing and Compounding of Polymer/Filler and Polymer/Polymer Mixtures with the Aid of Ultrasound. Rubber Chem. Technol..

[12] Kim K.J., VanderKooi J. (2004). Reactive Batch Mixing for Improved Silica-Silane Coupling. Int. Polym. Process..

[13] Kim S.M., Cho H.W., Kim J.W., Kim K.J. (2010). Effects of processing geometry on the mechanical properties and silica dispersion of silica-filled isobutylene-isoprene rubber (IIR) compounds. Elastomers Compos..

